# A new subspecies of *Trypanosoma cyclops* found in the Australian terrestrial leech *Chtonobdella bilineata*

**DOI:** 10.1017/S0031182021000639

**Published:** 2021-09

**Authors:** John Ellis, Joel Barratt, Alexa Kaufer, Lauren Pearn, Brigette Armstrong, Michael Johnson, Yasunori Park, Lara Downey, Maisie Cao, Levina Neill, Rogan Lee, Bethany Ellis, Kevin Tyler, Zhao-Rong Lun, Damien Stark

**Affiliations:** 1 School of Life Sciences, University of Technology Sydney, Broadway, NSW, Australia; 2 Department of Microbiology, St Vincent's Hospital, Sydney, Darlinghurst, NSW, Australia; 3 NSW Health Pathology, ICPMR, Westmead Hospital, Westmead, NSW, Australia; 4 Centre for Infectious Diseases and Microbiology Laboratory Services, ICPMR, Westmead Hospital, Westmead, NSW, Australia; 5 Research School of Earth Sciences, Australian National University, Canberra, ACT, Australia; 6 Norwich Medical School, University of East Anglia, Norwich, Norfolk, UK; 7 Center for Parasitic Organisms, State Key Laboratory of Biocontrol, School of Life Sciences, Sun Yat-Sen University, Guangzhou 510275, China

**Keywords:** Diversity, leech, phylogeny, swamp wallaby, *Trypanosoma*, *Trypanosoma cyclops*

## Abstract

Previously, it was suggested that haemadipsid leeches represent an important vector of trypanosomes amongst native animals in Australia. Consequently, *Chtonobdella bilineata* leeches were investigated for the presence of trypanosome species by polymerase chain reaction (PCR), DNA sequencing and *in vitro* isolation. Phylogenetic analysis ensued to further define the populations present. PCR targeting the 28S rDNA demonstrated that over 95% of *C. bilineata* contained trypanosomes; diversity profiling by deep amplicon sequencing of 18S rDNA indicated the presence of four different clusters related to the *Trypanosoma* (*Megatrypanum*) *theileri*. Novy–MacNeal–Nicolle slopes with liquid overlay were used to isolate trypanosomes into culture that proved similar in morphology to *Trypanosoma cyclops* in that they contained a large numbers of acidocalcisomes. Phylogeny of 18S rDNA/GAPDH/ND5 DNA sequences from primary cultures and subclones showed the trypanosomes were monophyletic, with *T. cyclops* as a sister group. Blood-meal analysis of leeches showed that leeches primarily contained blood from swamp wallaby (*Wallabia bicolour*), human (*Homo sapiens*) or horse (*Equus* sp.). The leech *C. bilineata* is a host for at least five lineages of *Trypanosoma* sp. and these are monophyletic with *T. cyclops*; we propose *Trypanosoma cyclops australiensis* as a subspecies of *T. cyclops* based on genetic similarity and biogeography considerations.

## Graphical abstract

**Figure:**
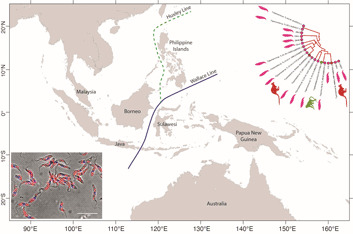

## Introduction

Trypanosomatids are flagellated protozoan parasites that infect a wide range of taxa and are ubiquitous across the globe (Kaufer *et al*., 2017). Some species are the aetiological agents of serious human diseases, such as African sleeping sickness and Chagas disease, and agriculturally important diseases, such as Surra and Nagana. Although their impact on other parts of the world has been known for a very long time, only recently has awareness of the great diversity of trypanosomes in Australia been considered (Thompson *et al*., 2014; Krige *et al*., 2019).

The terrestrial leech *Chtonobdella bilineata* is very common down the east coast of Australia and the presence of trypanosomes in *Chtonobdella* spp. was first reported in 1968 (Richardson and Hunt, 1968). Using molecular methods, Hamilton tested four species of leeches for the presence of trypanosomes and found them to be surprisingly common (Hamilton *et al*., 2005). That study suggested that leeches may be important vectors of trypanosomes in Australia, a sentiment initially proposed by Noyes *et al*. (1999) and recently endorsed by others (Averis *et al*., 2009; Cooper *et al*., 2017). The *Trypanosoma* species found in Australian terrestrial leeches also shared a close phylogenetic relationship with *Trypanosoma cyclops* and *Trypanosoma theileri* which is the type species of the subgenus *Megatrypanum* (Hamilton *et al*., 2005).

*Megatrypanum* comprises a group of large trypanosomes belonging to the Stercoraria section; trypanosomes whose life cycles are completed in the hindgut of an insect vector (Hoare, 1972). The *Megatrypanum* contain a kinetoplast situated close to the nucleus and away from the posterior end of the cell. They possess some morphological affinities with trypanosomes from reptiles and birds such as *Trypanosoma grayi* and *Trypanosoma avium*, which also possess similar life cycles, such that these species were once suggested to be part of the *Megatrypanum* (Hoare, 1972). The platypus-infecting *Trypanosoma binneyi* has a suspected leech vector (Paparini *et al*., 2014), and was once recognized as a member of the *Megatrypanum* subgenus based on host (mammalian) and morphological factors (e.g. kinetoplast situated near the nucleus, far from the cells anterior) (Hoare, 1972). The subgenus *Megatrypanum* is however now restricted to those large trypanosomes found in ungulates, such as *T. theileri* (Garcia *et al*., 2020).

This study sought to provide greater clarity on the relationship between terrestrial haemadipsid leeches and the diversity of trypanosomes found in them. We investigated the presence and diversity of trypanosomatids in the leech *C. bilienata* collected during volunteer bush care activities in and around Sydney, NSW, Australia. DNA barcoding was used to confirm the identity of the leech species, and *in-vitro* culture was used to isolate and propagate trypanosomatids from *C. bilineata* for subsequent characterization by light and electron microscopy. Polymerase chain reaction (PCR), DNA sequencing and molecular phylogenetic methods confirmed the relationship of these trypanosomes to other *Trypanosoma* species and more specifically to *T. cyclops*, a species found in Asian Macaques. The results obtained provide conclusive evidence that *C. bilineata* is a host for five lineages of *Trypanosoma* species and provides an on-going link between trypanosomes of Australian leeches and those found in the swamp wallaby.

## Materials and methods

### Leech collections

Leeches were collected by members of the local bush care community from predominantly Council-managed bush care sites (RR, latitude: −33.700469, longitude: 151.085419; OM, −33.699448, 151.092397; FP, −33.7035, 151.098; GMB, −33.591667, 151.300833), three of which (RR, OM, FP) are located close to a popular hiking trail into the Berowra Valley Regional Park, 20 km north-west of the Sydney Central Business District (CBD). GMB is located on Pittwater, 43 km north of the Sydney CBD; a final site W (−33.676205, 151.177241) is a privately managed bush care site in the suburb of Duffys Forest, 28 km north of the Sydney CBD. Under the Köppen climate classification, Sydney has a warm temperate climate (with a hot summer) where the mean annual rainfall is ~1077 mm (Terrey Hills weather station; close to Duffys Forest; −33.69, 151.23) and the mean average daily temperature is ~ 17°C.

### Confirmation of leech identity and detection of trypanosomes

DNA was extracted from leeches using the Genomic II DNA extraction kit (Bioline). DNA barcoding using the *COX1* gene was performed to assign leeches to a species. The primers LCO1490 (F, 5′ GGTCAACAAATCATAAAGATATTGG 3′) and HCO2198 (R, 5′ TAAACTTCAGGGTGACCAAAAAATCA 3′) were used to PCR amplify ~650 bp of the *COX1* gene (Folmer *et al*., 1994) at an annealing temperature of 51°C. For the detection of trypanosomes in leeches, the primers JT28F (5′ AGTGCAGATCTTGGTTGGC 3′) and JT28R (5′ GGTTCTCTGTTGCCCCTT 3′) were used to PCR amplify ~250 bp of the 28S rDNA at an annealing temperature of 57°C. PCR was performed with the MyTaq PCR kit (Bioline). Sanger sequencing of PCR products was performed by the service provider Macrogen (Korea). Searches of the NCBI sequence databases were performed using BLAST (https://blast.ncbi.nlm.nih.gov/Blast.cgi).

### MiSeq amplicon sequencing of leech DNAs

DNA from five leeches testing positive by PCR for the presence of trypanosomes were analysed by diversity profiling using targeted amplicon sequencing. Amplicon sequencing was performed by the service provider AGRF (http://www.agrf.org.au/). PCR amplicon sequencing consisted of two stages: conventional PCR amplification of the V4 region of the 18S rDNA using DNA extracted from leeches as template, followed by Illumina MiSeq Next Generation amplicon sequencing of the resulting amplicons. Illumina reads were quality trimmed using BBDuk Adapter/Quality Trimming Version 37.28, executed within Geneious Prime software (Build 2018-11-06 02:41, Biomatters, Ltd., Auckland, New Zealand). BBDuk parameters were set to remove all truseq, Nextera and PhiX adapters, to remove low-quality sequences from both read ends (using a minimum quality score of 20) and to discard short reads less than 100 bases long. Paired reads were then merged in Geneious using BBMerge Paired Read Merger Version 37.28 (using default parameters). Merged and unmerged reads were then mapped to an 18S rDNA reference sequence from *Trypanosoma cruzi* (GenBank accession: AF245382.1). Mapping parameters were set to allow a minimum overlap of 100 bases with 80% identity. A *de novo* assembly was then performed on reads that successfully mapped, using the Geneious assembler. Consensus sequences (i.e. contigs) were exported only if they achieved coverage of greater than 50 across the entire sequence. Variants were only separated if they obtained coverage of greater than 50 bases. If this was not the case, the contigs were collapsed into a single contig consensus sequence, with only the base with the highest coverage considered. The resulting contigs were assessed for their accuracy first by mapping the original reads (those that were *de novo* assembled) back to the resulting contigs, requiring 100% identity and an overlap of 50 bases; 1 base mismatch and 1 ambiguous base was tolerated. The contigs were subjected to a simple online BLASTN search to confirm that these contigs belong to trypanosomes based on BLAST similarity. This same approach was adopted for analyses of leech identity and their blood meals. For details, refer to Supplementary File 1.

### DNA barcoding of leech blood meals

To further investigate the source of the blood meal taken by leeches, PCR and DNA sequencing were performed using the primers L1085 (5′ CCCAAACTGGGATTAGATACCC 3′) and H1259 (5′ GTTTGCTGAAGATGGCGGTA 3′) (Kitano *et al*., 2007) that amplify a PCR product of ~215 bp from the 12S rDNA of vertebrates at an annealing temperature of 60°C.

### Isolation of trypanosomes into *in-vitro* culture

Initially, Novy–MacNeal–Nicolle (NNN) slopes containing antibiotics were overlaid with either 3 mL of Lockes solution, M3 or LIT (liver infusion tryptose) media. M3 medium contained 10% (v/v) heat-inactivated horse serum (Bovogen), isovitalex (20 mL L^−1^) and penicillin and streptomycin (Pen/Strep), but without haemoglobin (Barratt *et al*., 2017). LIT medium was made according to the ATCC 1029 medium recipe with modifications: liver broth (Oxoid, 9 g L^−1^), lab-lemco powder (Oxoid, 5 g L^−1^), NaCl (5 g L^−1^), Na_2_HPO_4_ (7 g L^−1^), glucose (1 g L^−1^) were added to DDW and sterilized by autoclaving. Ten percent heat-inactivated horse serum, Pen/Strep and laked horse blood (20 mL L^−1^) were added as a source of haemin (Lemos *et al*., 2013). Laked horse blood was prepared by adding an equal volume of DDW to horse blood (Serum Australis) and freeze/thawing it three times at 56°C. LIT was filtered through a 0.2 *μ*m filter before use. After much testing, M3-containing laked horse blood became routinely used as the preferred overlay for the NNN slopes.

Other slopes tried for the primary isolation of cultures with the same overlays were (1) modified sloppy Evans (MSE) (Noyes *et al*., 1999), (2) diphasic blood agar with 10% defibrinated horse blood (from Serum Australis) [using 4% oxoid blood agar base no. 2 in ATCC medium 449, page 599 (Atlas, 2010) (DPA10)], (3) Columbia blood agar with 15% laked defibrinated horse blood [page 439 (Atlas, 2010) (CBA15)] and (4) diphasic blood agar with 30% defibrinated horse blood [page 600 (Atlas, 2010) (DPA30)]. DPA30 contained 40 g L^−1^ Oxoid blood agar base no. 2, 10 g lab lemco powder, 10 g agar, 2.5 g L^−1^ NaCl, 1 g L^−1^ glucose and 30% defibrinated horse blood. Slopes 1–4 all contained Pen/Strep.

The presence of bacterial species (and their antibiotic sensitivities) in xenic cultures of trypansosomes was investigated by standard microbiological methods performed at St. Vincent's Hospital (Sydney) Microbiology Department.

Cloning of the trypanosomes was performed once by limiting dilution and visualization of a single cell in a single droplet of medium. Although these lines do not represent true clones, they are referred to here as subclones. Subclones were established in M3 medium containing laked horse blood plus 10% sterile spent medium from a current actively growing primary culture. After the first passage they were grown in M3 medium without the spent medium addition.

Once stable and after eradication of microbial contaminants by antibiotic treatment, all axenic cultures (primary and subclone populations) were weaned from the NNN slopes into M3 media (pH 7) containing 10% (v/v) heat inactivated horse serum (Bovogen), 10% (v/v) tryptone-phosphate broth, non-essential amino acid supplement (Gibco, 1 × final concentration) and haemin (2.5 *μ*g mL^−1^ final concentration, added from a stock solution containing 2.5 mg mL^−1^ 2% NaOH) in place of laked horse blood. Given the history of the cultures and the common microbial contaminants, gentamycin, amikacin and vancomycin were routinely added to the media.

Trypanosome cultures were frozen at −80°C in either M3 medium containing 10% DMSO or Triladyl containing 20% egg yolk emulsion (SR0047, Oxoid).

### Microscopy of cultured trypanosomes

Air-dried smears of trypanosome cultures (from NNN slopes with an M3 overlay) on glass slides were fixed in 100% methanol and stained using either a commercial Leishman or Giemsa stain. Slides were then washed quickly in tap water, air dried before mounting with a coverslip.

For the determination of cell sizes, cultured trypanosomes were stained with Hoechst 33347 (2 *μ*g mL^−1^) and Nile red (10 *μ*g mL^−1^) for 20 min at room temperature. Cells were then pelleted gently and fixed in 4% paraformaldehyde/phosphate buffered saline (PBS) for 20 min at room temperature, after which they were washed three times in PBS. A 20 *μ*L volume was placed on a poly-l-lysine coated coverslip and the cells spread thinly across the surface to allow attachment to the coverslip. Cells were left to attach for 10 min and the coverslip was then placed onto a slide with 10 *μ*L of mounting media (90% glycerol containing 0.5% *N*-propyl gallate). Cells were imaged on an Olympus BX51 microscope using a 100× NA 1.3 Plan Fluor objective lens calibrated for distance measurements. For transmission images, differential interference contrast (DIC) was used. For fluorescence, Hoescht 33342 was detected using an Olympus U-MWU filter cube (ex: 330–385 nm, em: 420 nm LP). Nile red was detected using a U-MWIG2 filter cube (ex: 520–550 nm, em: 580 nm LP). Images were collected by using an Olympus DP73 camera. Cell measurements were performed with freehand lines combined with the ‘Measure’ function in FIJI (Schindelin *et al*., 2012).

### Electron microscopy

Cultured trypanosomes (from NNN slopes with an M3 overlay) were examined using the encapsulation method (Lazzaro, 1983). Briefly, trypanosomes were fixed in 2.5% glutaraldehyde in 0.1 m cacodylate buffer (pH 7.4) for a minimum of 1 h, after which the cells were washed extensively in cacodylate buffer, before being resuspended in bovine serum albumin (BSA; fraction V) for 20 min. The BSA was then replaced with fresh modified Karnovsky fixative. The tubes were then incubated upright at 2–8°C for at least 5 h to allow cross linking. The tube was then cut with a fresh single edge razor blade and the BSA-encapsulated pellets were removed. The pellet was cut into blocks of approximately 1 mm thickness and processed using a traditional method for preparation of solid tissue blocks for transmission electron microscopy (TEM) (Glauert, 1974). Specimen blocks were post fixed in 2% osmium tetroxide in 0.1 m cacodylate buffer, rinsed in distilled water and transferred to 2% uranyl acetate for 1 h. They were dehydrated in an ethanol series (10 min changes of each), 50% through to 100% ethanol, then transferred to acetone. Blocks were infiltrated with acetone/resin mixture for 1 h followed by 3× 10 min changes of 100% TAAB TLV soft resin epoxy at 70°C. Blocks were embedded in TAAB TLV soft resin and Polymerize resin at 70°C for 10 h. Blocks were sectioned on a Leica ultramicrotome (UC6RT), using a diamond knife and the 80–90 nm ultrathin sections produced were collected onto copper grids. They were post stained with 2% (ethanolic) uranyl acetate followed by Reynolds Lead citrate. Specimen was examined at 80 kV on a Hitachi HT7800 120 kV Transmission Electron Microscope. Images were captured using an AMT NanoSprint 12 MP CMOS camera.

### Construction of phylogenetic trees

DNA was extracted from cultured trypanosomes using a Qiagen EZ1 robot. PCR of the 18S rDNA was performed using the primers TRY927F (5′ GAAACAAGAAACACGGGAG 3′) and TRY927R (5′ CTACTGGGCAGCTTGGA 3′) (Noyes *et al*., 1999, 2000). Sanger sequencing of these amplicons was performed twice in each direction by the service provider Macrogen. Low-quality bases at either end of the PCR product were manually trimmed in the Geneious Prime interface, and the trimmed sequences were merged into a single contig that was used in BLAST to search for similar sequences. PCR and DNA sequencing of part of the GAPDH gene (gGAPDH) was conducted using primers GAPTRY-modF (5′ GGBCGCATGGTSTTCCAG 3′) and GAPTRYrR (5′ CCCCACTCGTTRTCRTACC 3′) (Borghesan *et al*., 2013). A search of overlapping gGAPDH sequences from *Trypanosoma* sp. was performed against the NCBI nucleotide database and several were downloaded, focusing on sequences from *T. cyclops*, *T theileri* and several trypanosome species identified previously from Australian mammals. Sequences were manually trimmed to the same length as those generated from the leech trypanosomes in this study. Analyses of the ND5 region of the maxicircle was performed by PCR and DNA sequencing of the PCR products using primers ND5-1F (5′ GAGAAACTTATTTGGCAT 3′) and ND5-1R (5′ CRGCGTGGATTAATGCRGATAC 3′) that amplify ~900 bp at an annealing temperature of 55°C. The resulting sequences were used to construct phylogenies using the ‘ape’ and ‘phangorn’ R packages.

Sequence concatenation of the 18S rDNA and gGAPDH was manually performed using Geneious Prime. Alignments were performed in Geneious using Muscle (version 3.8.425) and manually curated where necessary. The alignment was exported from Geneious in fasta format and genetic distances calculated using the Phangorn package in R. A maximum likelihood tree was constructed as described above for gGAPDH. Accession numbers of sequences used in this study (for sequences not from Australian leeches): (1) for 18S rDNA – *Trypanosoma* sp. H25: AJ009168, *T. cyclops*: AJ131958.1, *Trypanosoma* sp. wallaby ABF: AJ620564.1, (2) for gGAPDH – *Trypanosoma* sp. H25: AJ620276.1, *T. cyclops*: the current study, *Trypanosoma* sp. wallaby ABF: AJ620278.1.

## Results

### Detection of trypanosomes in *C. bilineata*

Fifty leeches were barcoded using the *COX1* gene for leech identification. DNA sequence data from them confirmed them all as *C. bilineata*. Of the 80 leeches tested, 75 tested positive for the presence of trypanosomes by PCR using primers JT28F and JT28R which amplify the 28S rDNA. BLASTN searches with sequences derived from these PCR products gave hits to a variety of trypanosome species.

### MiSeq DNA sequencing

Using MiSeq data generated from the V4 region of the 18S rDNA, a contig was obtained for each of the five leeches (BA3, BA19, BA32, BA77 and BA78), which was identical to the original reference sequence KT592372.1 thereby confirming the leeches were *C. bilineata*. An NCBI blast search showed that other vertebrate sequences were present in the MiSeq data that could be derived from any vertebrate, including humans, cats, deer, pigs and a variety of monkeys (not native in Australia). A blood-meal result could not be obtained from BA3 or BA32.

The MiSeq data generated from the V4 region supported the presence of *Trypanosoma* spp. 18S rDNA sequences (114 bp in length) in every leech sample (BA3, BA19, BA32, BA77 and BA78). These contigs gave BLASTN hits to sequences from *T. theileri* and *T. cyclops*.

### Blood-meal analyses using 12s rDNA

Blood-meal analysis of 31 leeches using 12S rDNA identified three main vertebrate species by BLASTN searching of sequences from the PCR products obtained: swamp wallaby (*Wallabia bicolour*) (*n* = 11), horse (*Equus* sp.) (*n* = 12) and human (*Homo sapiens*) (*n* = 8).

### Parasite culture

Eleven primary cell cultures were initially obtained (from 11 different leeches) using NNN slopes with overlays (one with Lockes, two with LIT and eight with M3). None of the other slopes (DPA10, CBA15, MSE and DPA30) or media tested (e.g. M3 without a slope) were successfully used for the primary isolation of trypanosomes from *C. bilineata*. These results suggested that NNN slopes with an M3 overlay was more likely to result in a successful isolation of trypanosomes than LIT or Lockes and so the combination of NNN with an M3 overlay was subsequently adopted for routine use.

The antibiotics Pen/strep, gentamycin and vancomycin were used to control the majority of bacterial species found in cultures, although yeast and mucoid gram negative rods (not speciated by routine hospital microbiology methods) were common contaminants. Two bacterial species present in long-term cultures (*Brevundimonas diminuta* and *Stenotrophomonas maltophilia*) were subsequently treated and removed with amikacin and cotrimoxazole. Given the presence of un-identified yeast in several of the cultures, amphotericin B (Thermofisher Antibiotic-Antimycotic cat. no. 15240062) or Fungin (https://www.invivogen.com/fungin) were eventually included in the culture media at the recommended concentrations for prevention of contamination (10 *μ*g mL^−1^). Higher concentrations were lethal to the trypanosomes. Despite the incorporation of these anti-mycotic reagents several of the cultures succumbed to heavy yeast/fungal infections (e.g. OM1, RR2, RR3, GMB2 and 1A) despite being maintained for several months.

**Fig. 1.**
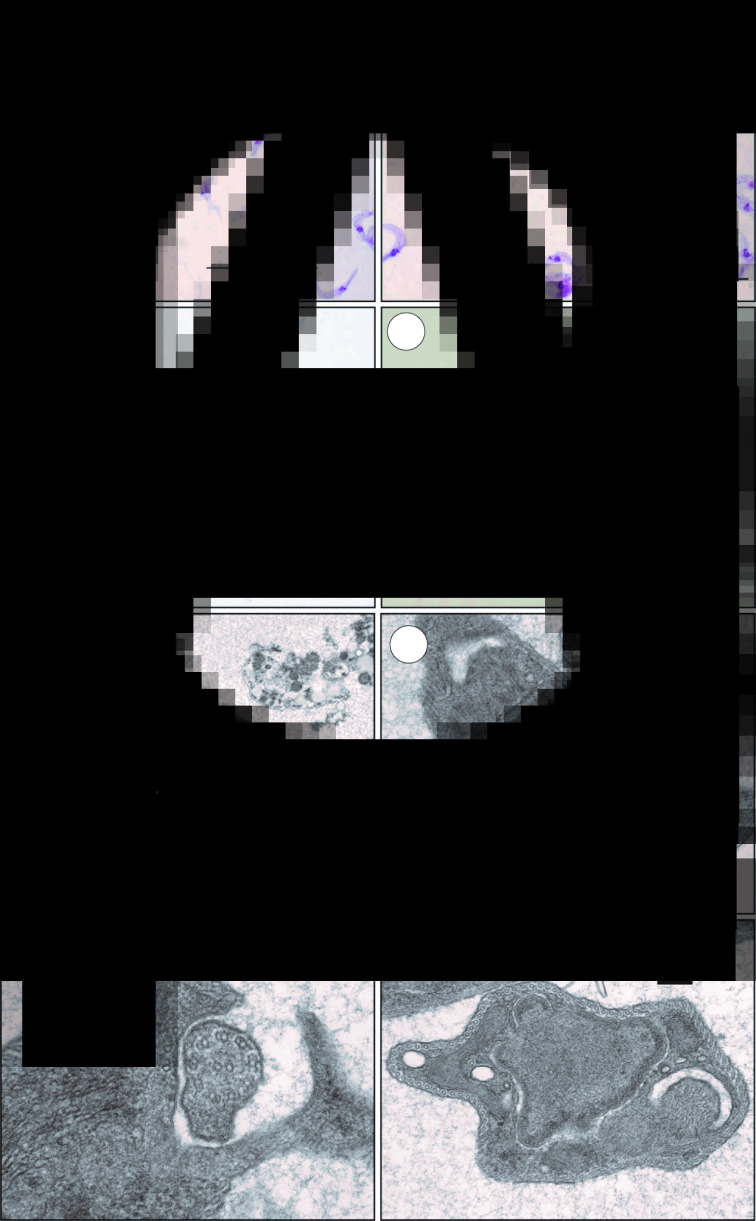
Light and electron microscopy of trypanosomes in the LIT2 culture. Light microscope images of Giemsa-stained trypanosomes. Panels (a) and (b) show primarily the epimastigote forms with a centrally located nucleus and kinetoplast in close proximity; panel (c) shows a trypomastigote and an epimastigote for size comparison; panel (d) shows other commonly seen developmental stages. Scale bar represents 40 *μ*m. TEM images show dense vacuolation of the trypanosome cytoplasm (pale grey densities are consistent with lipid, clear white areas are more in keeping with vacuoles) (e), presence of kinetoplast (f), cross-section of flagellum bearing flagella sheaf in flagella pocket (g) and cross-section of body showing micro-tubules beneath a unit cell membrane and a nucleus with peripheral chromatin.

Stained smears of the trypanosome cultures revealed a diversity of morphological types present in the cell cultures (Fig. 1). The most common form seen is an epimastigote with a nucleus that is normally centrally located in the body of the trypanosome or slightly posteriorly located. The kinetoplast is seen close by in stained specimens examined by light microscopy (panels a and b), more commonly located on the posterior side of the nucleus. The posterior end is either slightly rounded or tapered to a fine point. The body length (not including the flagellum) varied significantly but was typically 19–27 *μ*m long and 3 *μ*m at its widest point. Other morphological types in culture were less common; including trypomastigotes with a kinetoplast located close to the posterior end (panel c) and other developmental forms (panel d). Dividing forms were commonly seen through the characteristic V-shape adopted during division which is observed in other trypanosome species (panel e).

TEM showed the cytoplasm of the epimastigote forms to be highly vacuolated with large numbers of acidocalcisomes (Fig. 1e, f); the nucleus contains a central nucleolus and condense chromatin at the periphery attached to the nuclear membrane. The kinetoplast was also proximal to the nucleus (panel f). The flagellum has the classical ‘9 + 2’ arrangement of microtubules (panel g). The width of the cultured trypanosomes (LIT culture) was in the range of 3.5–3.9 *μ*m, which is slightly larger than that estimated by light microscopy. TEM confirms the presence of sub-membranous microtubules and an enclosing unit membrane (panel h).

**Fig. 2.**
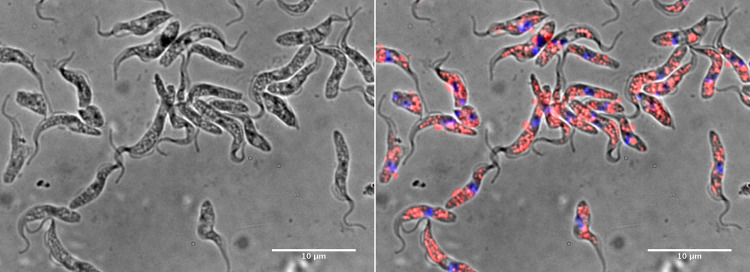
Hoechst 33342 and Nile red-stained trypanosomes from the LIT2 culture. Left panel: DIC image showing typical culture forms that contain distinct light coloured granules that are ubiquitously spread through the cell. Right panel: fluorescence staining with Nile red localizing within the light coloured granules. The kinetoplast stains brightly in blue, whilst the nucleus is stained in blue but is less bright.

Figure 2 shows a typical primary culture (called LIT2) stained with DAPI and Nile red; the pattern of Nile red staining suggests that lipid granules are widely distributed and highly abundant through the cells. Size measurements for two cultures (LIT2 and its subclone LIT2C7) stained in this way are shown in Table 1; the two cultures differ slightly in their width (significant by *t* test at <0.0001). The primary culture LIT2 is slightly thinner than the subclone derived from it.

**Table 1.** Size measurements of trypanosomes from the LIT2 primary culture and the LIT2C7 subclone culture

	PN (*μ*m)	NA (*μ*m)	KN (*μ*m)	PN/NA	PN/KN	PN + NA (*μ*m)	F (*μ*m)	W (*μ*m)
LIT2	3.4	4.9	0.5	0.7	7.3	8.4	3.5	1.1
	3.3	5.3	0.8	0.6	4.2	8.6	3.8	1.5
	4.3	6.1	0.4	0.7	11.5	10.4	3.4	1.7
	3.6	4.9	2	0.7	1.8	8.5	2.7	1.5
	2.2	5.9	0.4	0.4	5.8	8.1	2.6	1.5
	4.1	4.1	0.4	1	10	8.2	5.1	1.4
	2.7	5.9	1.1	0.5	5.9	8.6	2	1.5
	4	4.5	0.7	0.9	5.5	8.5	3.1	1.4
	3.8	5.3	0.6	0.7	5.9	9.9	2.8	1.1
	2.9	5.1	0.9	0.6	3.3	8	2.6	1.3
Mean	3.4	5.2	0.8	0.7	6.1	8.7	3.159	1.4
s.d.	0.7	0.6	0.5	0.2	2.9	0.8	0.862	0.2
LIT2 C7	3.5	6.2	−0.4	0.6	NA	9.6	7.3	3.8
	5	6.2	0.9	0.8	6.2	11.2	7.4	4.8
	4.3	4.6	0.7	0.9	4.6	8.8	NA	3.4
	3.6	9.2	0.4	0.4	9.2	12.8	NA	5.7
	4.8	7.6	1.3	0.6	7.7	12.4	NA	4.6
	3.9	6.6	−0.2	0.6	NA	10.5	NA	4
	2	4.5	−0.5	0.4	NA	6.5	4.6	2
	3.7	4.5	−0.1	0.8	NA	8.2	4	2.8
	2.6	4.3	−0.3	0.6	NA	6.9	3.5	1.8
	2	6.6	0.3	0.3	6.7	8.6	NA	4.5
Mean	3.5	6	0.72	0.6	6.9	9.6	5.4	3.7
s.d.	1.1	1.6	0.4	0.2	1.7	2.2	1.9	1.3

PN, posterior to middle of nucleus; NA, anterior to middle of nucleus; KN, kinetoplast to nucleus along the posterior anterior axis. A negative KN number is due to the kinetoplast being on the anterior side of the nucleus; PN/NA, nuclear index (<1 means the nucleus is located in the posterior); PN/KN, kinetoplast index (>2 indicates that kinetoplast is located closer to the nucleus than the posterior); PN + NA, cell length (not including flagella); F, length of flagella; W, width of cell; NA, not determined.

### Phylogenetic analyses

**Fig. 3.**
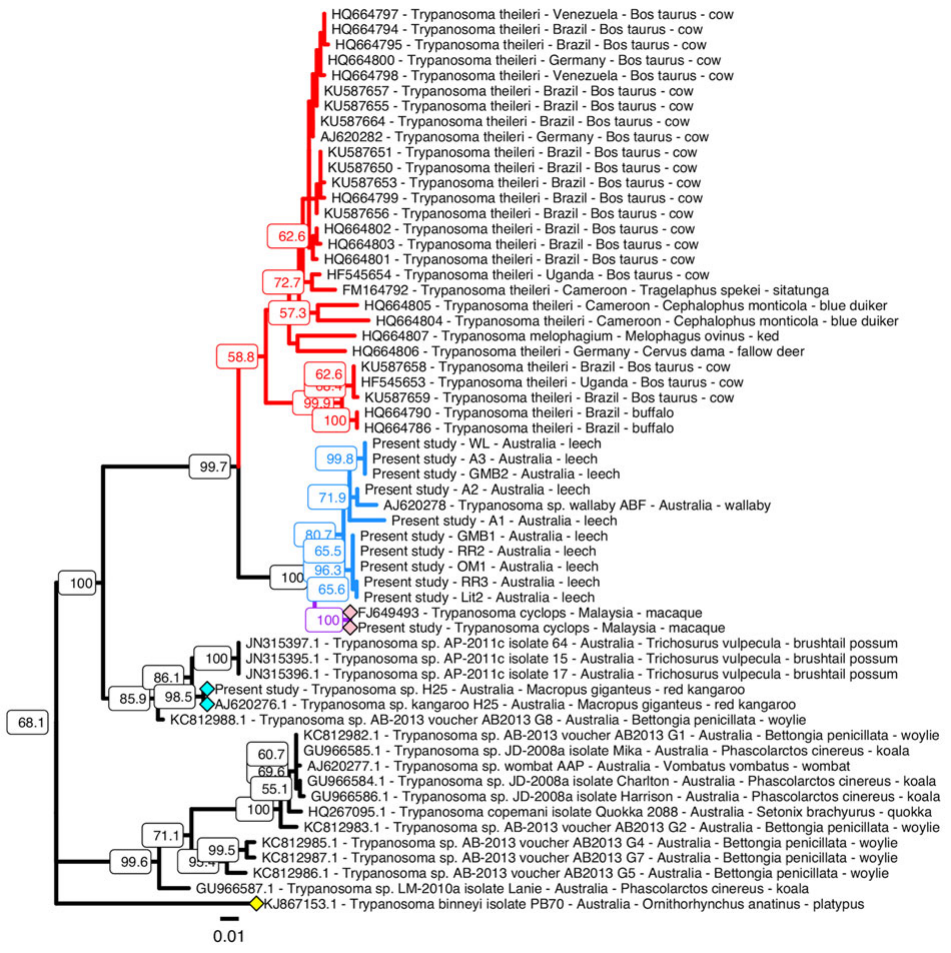
Maximum-likelihood phylogeny of gGAPDH sequences of various trypanosomes. A 487 base pair fragment of the gGAPDH gene from 78 trypanosome species was aligned using MUSCLE (version 3.8.425), and the phylogeny shown was generated from this alignment using the ‘Phangorn’ package in R. Distances were first calculated using the dist.ml function and a minimum evolution tree was constructed using the fastme.bal function from the ‘ape’ R package. A maximum-likelihood tree was optimized using the pml and optim.pml functions, applying the NNI rearrangement model (log-likelihood: −3110.909). The bootstrap.pml function was used to calculate non-parametric bootstrap values across 1000 samples. Only bootstrap values above 55 are shown. *Trypanosoma binneyi* isolate PB70 (yellow diamond) was included as an outgroup. Blue diamonds = *Trypanosoma* sp. H25, pink diamonds = *Trypanosoma cyclops*. The red clade contains all *Trypanosoma theileri* isolates included in this analysis, and the blue clade contains all *Trypanosoma* sequences obtained from leeches collected in this study. These leech trypanosomes (blue branches) form a well-supported clade with *T. cyclops* (purple branches), with 100% bootstrap support. Scale bar represents the number of substitutions per site. GenBank accession numbers are included at the beginning of each sequence name followed by the *Trypanosoma* species (and/or strain/isolate), the country of origin and the host from which the trypanosome was isolated. This phylogeny was annotated using the ggtree package (R).

A maximum likelihood tree constructed from GAPDH sequences from a range of trypanosome species (Fig. 3) indicated the leech trypanosomes from this study form a single clade that also contained *T. cyclops*, a species isolated from Malaysian macaques (Weinman, 1972; Heywood *et al*., 1974). This clade also contained *Trypanosoma* sp. ABF, previously isolated from a swamp wallaby (Hamilton *et al*., 2005), closely associated with those trypanosomes isolated from *C. bilineata* in the current study. The *T. cyclops* clade containing the leech trypanosomes isolated in this study formed a sister group to the *T. theileri* clade.

**Fig. 4.**
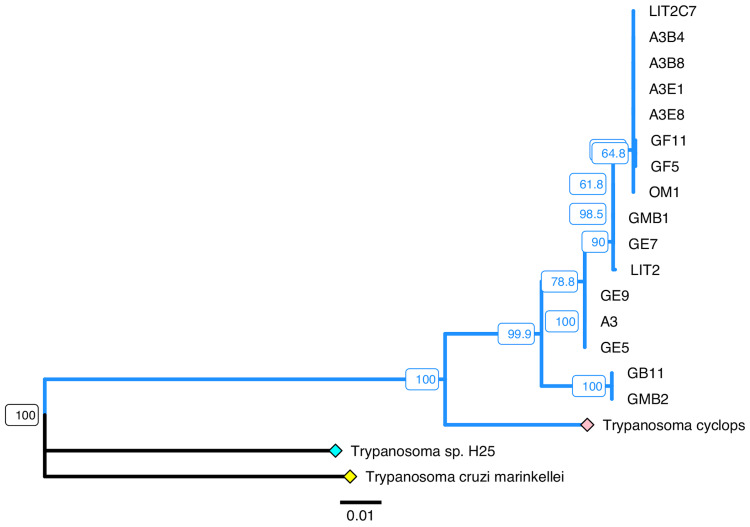
Maximum-likelihood phylogeny of concatenated sequences (18S rDNA, gGAPDH and ND5) of trypanosomes from *Chtonobdella bilineata*. An approximately 2100 base pair sequence (species-dependent) was constructed by artificial concatenation of partial 18S rDNA (~870 bp), gGAPDH (487 bp) and ND5 (~750 bp) sequences of 19 trypanosomes, concatenated in that order. These sequences were aligned using MUSCLE (version 3.8.425), and the phylogeny shown was generated from this alignment using the ‘Phangorn’ package in R. Distances were first calculated using the dist.ml function and a minimum evolution tree was constructed using the fastme.bal function in the ‘ape’ R package. A maximum-likelihood tree was optimized using the pml and optim.pml functions, applying the NNI rearrangement model (log-likelihood: −6212.942). The bootstrap.pml function was used to calculate non-parametric bootstrap values across 1000 samples. Only bootstrap values above 55 are shown. *Trypanosoma cruzi marinkellei* (yellow diamond) was included as an outgroup. Blue diamond = *Trypanosoma* sp. H25, pink diamond = *T. cyclops*. The blue clade contains all *Trypanosoma* sequences obtained from *C. bilineata* collected in this study which along with *T. cyclops*, form a single well-supported clade with 100% bootstrap support. Scale bar represents the number of substitutions per site. Sequences of *Trypanosoma* sp. H25, *T. cyclops* and all leech trypanosomes were generated in this study. The concatenated sequence of *T. cruzi marinkellei* was generated by concatenating the 18S rDNA sequence from GenBank accession (GBA) AJ009150.1 (bases 796–1666), the gGAPDH sequence from GBA FJ649495.1 (bases 157–643) and the ND5 sequence from GBA KC427240.1 (bases 1218–1975). This phylogeny was annotated using the ggtree package (R).

The maximum likelihood tree of concatenated 18S rDNA, GAPDH and ND5 also supported the existence of a very close phylogenetic relationship between *T. cyclops* and the trypanosomes obtained from leeches in this study (Fig. 4). This tree contained five clades of trypanosomes from *C. bilineata*; it also highlights the fact that the primary cultures obtained are mixed cultures as primary cultures (LIT2, GMB1 and A3) and their subclones (LIT2C7, GF11, GF5, GE5, GE9, GB11, A3B4, A3B8, A3E1 and A3E8) appear in different clades.

For the ND5 locus alone, analyses support three ND5 sequence types and two main clusters of trypanosome maxicircle within these populations. GF11 and GF5 differ from other members of a larger cluster by a single nucleotide polymorphism and the smallest cluster contains only GMB2 and GB11. GB11 was derived from the GMB1 primary culture which appears in the other clade.

### Comparisons to *T. theileri*

A question remains over the identity and genetic diversity of the trypanosomes found in *C. bilineata* – are the lineages present representative of one or more different species? Although it is difficult to determine this with the cultures available, we made comparisons of the genetic distance data derived from these trypanosomes to those of *T. theileri* which is a common *Megatrypanum* found in ungulates (Rodrigues *et al*., 2006). It is also very closely related to the *T. cyclops* clade (Fig. 3). Concatenated SSU rDNA and gGAPDH gene sequence alignments were constructed which included the *Megatrypanum* species *T. theileri*, as well as *T. cyclops*, and the Australia trypanosomes *Trypanosoma* sp. ABF and *Trypanosoma* sp. H25 described previously.

Genetic distance data were generated from these sequence alignments and the distance data from the *T. cyclops* clade compared to the two main lineages of *T. theileri*. A summary of the distance analyses is shown in Table 2. The genetic distances between the trypanosomes found in *C. bilineata* and *T. cyclops* at the 18S rDNA and GAPDH loci analysed is significantly less than the distances found amongst various isolates of *T. theileri*. The clade containing *T. cyclops* and the trypanosomes from leeches are therefore all very closely related to each other, suggesting that they may be of the same related species. In comparison, the relationship between the two *T. theileri* genetic lineages is also very low. The fact that the distances within the *T. theileri* cluster are greater than those observed within the *T. cyclops* clade suggests that the trypanosomes in *C. bilineata* should be considered as *T. cyclops* or *T. cyclops*-like.

**Table 2.** Summary of genetic distances determined for *Trypanosoma theileri* and the *Trypanosoma cyclops* group

	*T. theileri*	*T. cyclops* group^a^	*T. theileri*	*T. cyclops* group^a^
	18S rDNA	18S rDNA	GAPDH	GAPDH
Maximum distance	0.0130	0.0019	0.1029	0.0346
Average distance	0.0055	0.0002	0.0366	0.0050
Standard deviation	0.0039	0.0006	0.0292	0.0104

a Includes *T. cyclops* and *Trypanosoma* from *C. bilineata*.

## Discussion

In this study, we investigated the diversity of trypanosomatids in *C. bilineata* leeches collected from several locations on the outskirts of Sydney, Australia. PCR and DNA sequencing showed that nearly all the leeches studied contained trypanosomes, confirming the observations of Hamilton and colleagues that they are very common infections (Hamilton *et al*., 2005). Trypanosome cultures were established from leeches using NNN slopes with a liquid overlay. The M3 overlay was superior to the others that were tried for isolation of trypanosomes, including Lockes and LIT media. The use of other slope types for isolation and culturing of trypanosomes, including those related to chocolate agar, was unsuccessful.

Microscopy showed that the trypanosomes in culture at 25°C were very similar in morphology and structure, as viewed by light and electron microscopy, to those of *T. cyclops* (Weinman, 1972; Heywood *et al*., 1974). On blood slopes at 25°C, *T. cyclops* proliferates as epimastigote and related forms, and the morphology of the trypanosomes cultured here is strikingly similar to *T. cyclops*; for example, the epimastigotes have the kinetoplast located close to the nucleus; the cytoplasm is highly vacuolated with acidocalcisomes and the nucleus contains a central nucleolus with condensed chromatin in contact with the nuclear membrane.

However, there are several reported phenotypic characteristics of *T. cyclops* that are different from the parasites described in this study. Firstly, the original report suggests a much larger size for cultured forms of *T. cyclops*; up to 45 *μ*m (Weinman, 1972). Secondly, *T. cyclops* grown in the presence of haemoglobin become pigmented (Heywood *et al*., 1974). Neither of these characteristics was seen in trypanosomes cultured from *C. bilineata*. One feature they do have in common however is the highly vacuolated cytoplasm, comprising vacuoles that are either lipid-containing reservosomes (Pereira *et al*., 2011) or acidocalcisomes that have been assigned a range of functions, including osmoregulation and blood coagulation (Moreno and Docampo, 2009; Docampo and Huang, 2016). The TEM densities of these vacuoles are more akin to acidocalcisomes than reservosomes, and the Nile red staining of neutral lipids shows the presence of numerous lipid-containing vacuoles. Why these trypanosomes possess such a large number of acidocalcisomes is a mystery. Terrestrial leeches are commonly found in wet, humid ecosystems and their visibility and abundance is typically associated with precipitation (Tan and Liang, 2000; Drinkwater *et al*., 2020). During dry periods terrestrial leeches are believed to borrow into the ground and lie dormant, and it was particularly noticeable during this study that *C. bilineata* was uncommon during the dry months on the year. A large number of acidocalcisomes may represent one adaptation that *T. cyclops* and the trypansosomes of *C. bilineata* have evolved to allow persistence during drier times when water is scarce. Alternatively, they may play a role in adaptation to the processes associated with processing of the leech blood meal that is known to be retained for long periods of time.

Phylogenetic analyses of the trypanosome cultures showed that all cultured trypanosomes belonged to a clade associated with *T. cyclops* and more widely *T. theileri*, the type species of the subgenus *Megatrypanum*. Several recent studies have shown that other species also belong to this clade, including *Trypanosoma cervi*, *Trypanosoma melophagium* and other *T. theileri*-like isolates (Rodrigues *et al*., 2006; Martinkovic *et al*., 2012). All these are known to be present in *artiodactyla* ruminants such as cattle, sheep and deer. A previous study involving the isolation of trypanosomes from Australian marsupials (Hamilton *et al*., 2005) was significant as it extended the type and number of species belonging to the *T. cyclops* clade. Members of *Megatrypanum* are generally believed to be non-pathogenic (Calzolari *et al*., 2018).

The relationship of the *T. theileri* clade (*Megatrypanum*) to that containing *T. cyclops* is of particular interest. *Trypanosoma cyclops* was one of several unknown trypanosome species isolated from the Malaysian macaques, *Macaca nemestrina* and *Macaca ira* (now called *Macaca fascicularis*), at a time when studies were focussed on investigations into the presence of *T. cruzi* in southeast Asia, as well as the presence of a small number of cases of unexplained human trypanosomiasis (Weinman and Wiratmadja, 1969; Weinman, 1970). *Trypanosoma cyclops* was the only new species to emerge from those studies (Weinman, 1972), although *Trypanosoma conorhini* was also recognized at this time as a parasite of macaques (Cross *et al*., 1983; Deane *et al*., 1986) which appear to contain several un-defined species (Weinman, 1977). These two species, along with *T. cruzi* (Hodo *et al*., 2018), represent just a few of the trypanosome species known to exist in nonhuman primates (Ziccardi *et al*., 2000). More recently, *T. cyclops*-like trypanosomes were identified by sequence surveys in a variety of rodent species from Sulawesi, Indonesia (Winterhoff *et al*., 2020) and in Tasmanian Devils from Australia (Egan *et al*., 2020). From an historical perspective, the clade containing *T. cyclops* and *T. theileri* was first reported by Stevens *et al*. (1998) who suggested that the ‘ability to infect primates had evolved independently (presumably in Asia) from species in either of the two clades containing human infective trypanosomes’. These studies demonstrated that *T. cyclops* was ancestral to the *T. theileri* clade; indeed Hamilton also recognizes the *T. cyclops* clade within a much larger clade with *T. theileri* (Hamilton *et al*., 2005). The ancestral position of *T. cyclops* has also appeared in other recent studies as well (Rodrigues *et al*., 2006; Martinkovic *et al*., 2012).

Our analyses of genetic distances within the *T. cyclops* clade and their comparison to those of *T. theileri* show that the genetic diversity amongst trypanosomes in the *T. cyclops* clade is comparatively low. Indeed, the genetic distances amongst the leech trypanosomes and *T. cyclops* are comparable to or less than that found within *T. theileri*. Similarly, the diversity amongst *Trypanosoma dionisii* from bats, determined using the 18S rDNA, gGAPDH and Cytb genes was up to 6% (Barros *et al*., 2020), which is greater than the diversity observed amongst the trypanosomes found in *C. bilineata* in this study. Hence, we propose that the trypanosomes found in leeches in the current study should be considered as *T. cyclops* or a variant thereof. Consideration of the biogeography below leads us to propose the sub-species taxonomic designation of *Trypanosoma cyclops australiensis* for the trypanosomes found in *C. bilineata*. Following conventions for naming a subspecies (Winston, 1999), we therefore also recommend the use of *T. cyclops cyclops* as the nominotypical subspecies, representing the original isolates from Macaques (Weinman, 1972).

From a biogeographical viewpoint, the association of Australian trypanosomes into a clade with *T. cyclops* allows us to raise evidence on the historic association of southeast Asia and Australia in Gondwanaland. The time points of the breakup of Gondwana have been used in many studies to calibrate the evolutionary timescale of many different taxa including the trypanosomatids (Barratt *et al*., 2017). Studies indicate that SE Asia, including the Malaysian peninsula and Borneo were attached to the northwest coast of Australia in eastern Gondwana (Ridd, 1971; Audley-Charles *et al*., 1988; Burrett *et al*., 1991; Zahirovic *et al*., 2014), until its breakup over the Palaeozoic (*c*. 400 Ma) and Mesozoic eras (*c*. 145 Ma) (Turner *et al*., 2001; Hall, 2013; Metcalfe, 2017).

The biogeography of the Indo-Australian archipelago includes the Wallace's Line that demarks the boundary of the Asian and Australian types of biota (Camerini, 1993; Sarkar, 1998). Recent studies on the phylogeny and biogeography of macaques show that this lineage of animals appeared around 9 Ma (Disotell and Tosi, 2007), and their diversification and dispersal throughout southeast Asia is much more recent, occurring in a west to east direction that has recently transgressed the Wallace Line (Evans *et al*., 2020). It would therefore appear that trypanosome infections of macaques in southeast Asia are a relatively recent acquisition in evolutionary terms and consequently the phylogenetic relationship between *T. cyclops* and the trypanosomes of *C. bilineata* appear un-related to these events. Such considerations lead us to speculate that the evolution of the *T. cyclops* clade probably began before the breakup of Gondwanaland and so may well represent an example of vicariant speciation. Hence our suggestion for a subspecies called *Trypanosoma cyclops australiensis* as the taxonomic description for the populations of parasites found in *C. bilineata* of Australia. This suggestion is also founded on the hypothesis that *T. cyclops* is likely to be found in an unknown species of Asian leech that represent historically the primary host for this species. In his original description of *T. cyclops*, Weinman declared the vector of this species was unknown and that reduviids were refractory to infection (Weinman, 1972).

Borda and colleagues considered the phylogeny and evolutionary biogeography of haemadipsoid leeches in two relevant studies. In the first instance, they pointed out that *Chtonobdella* and the *Haemadipsidae* of southeast Asia must have had a common ancestor in Gondwana (Borda *et al*., 2008), giving rise to the extant species that we know today both in Australia and Southeast Asia. In a subsequent study, an ‘out of Asia’ proposal was made for their origins based on the idea of a trignathous ancestor, that was present in those parts of Gondwana that would eventually become SE Asia (Borda and Siddall, 2011). However, a complex story of dispersal and vicariant events were needed to explain the currently recognized distribution of the *Haemadipsidae*. Interestingly, long distance dispersal of *Chtonobdella palmyrae* was recently reported by procellariiform *seabirds around Japanese islands* (Nakano *et al*., 2020).

An important part of this history is the role of mountain ranges in Malaysia, Sumatra and Borneo acting as refugia for rainforests, during periods of significant environmental change that has occurred during the many glacial cycles (Lohman *et al*., 2011). Over time, the regions land and forest areas have changed significantly in response to sea levels and the largest biogeographical feature in the region known as the Sunda plains is presently under water, although this was not always the case (Woodruff, 2010; Mason *et al*., 2018). We note that moist environments are the preferred habitat for terrestrial leeches and it is quite possible that rainforests have acted as a refuge for both leeches and their trypanosomes during evolutionary time spans that have seen major changes to the landscape and climate.

Invertebrate-derived DNA (iDNA) is increasingly being used in studies on wildlife and to potentially survey populations (Schnell *et al*., 2015). A recent study surveyed leeches in legacy collections from China, Cambodia and Bangladesh using iDNA and identified they were feeding on a wide range of mammals, birds and reptiles including *Macaca* (Siddall *et al*., 2019). The same study also identified the presence of four clades of trypansosomes in *Chtonobdella tanae* which were closely related to *T. theileri* and *T. cyclops*. Our study generated a sequence dataset from *C. bilineata* using a MiSeq amplicon sequencing approach that also gave rise to four contigs that clustered ancestrally to the *T. theileri* clade. The data presented here are consistent with the observations of Siddall and colleagues, although our additional studies provide evidence for five clusters of closely related trypanosomes in *C. bilineata*.

iDNA was also used in this study to investigate previous blood meals of *C. bilineata*, as leeches retain blood for many months. Blood-meal analyses suggest that the *C. bilineata* leeches were feeding on a number of vertebrate species, including the swamp wallaby which is reasonably common in NSW, Australia. The previous isolation of the ABF culture by Hamilton *et al*. (2005) from swamp wallaby along with the isolation of genetically related cultures from *C. bilineata* in this study suggests that a natural cycle of transmission may be occurring between them. The swamp wallaby is seemingly a host for several species of trypanosomes (Thompson *et al*., 2014; Ortiz-Baez *et al*., 2020), including *Trypanosoma copemani* whose vector was proposed to be an *Ixodes* tick (Austen *et al*., 2011; Krige *et al*., 2019). In the study presented here, no evidence was found for *T. copemani* in *C. bilineata*.

However, based on other reports it is currently impossible to rule out other vectors of trypanosomes in the transmission to wallabies, including tabanids and sandflies. An Australian tabanid species is known to feed on macropods including wallaby (Muzari *et al*., 2010). However, the high prevalence of trypanosomes in *C. bilineata* does suggest infection is occurring at a very high rate. It is clearly feasible that the high incidence of trypanosomes in *C. bilineata* may be simply because of their predatory life cycle or other mechanisms of persistence that are related to the obscure leech life cycle. Animals, however, do appear at risk of a trypanosome infection being passed to them by the blood-feeding activities of leech species. It remains unknown whether trypanosomes found in terrestrial leeches are transmitted to animals during a leech bite, however we note that increasing evidence implicate aquatic leeches as a vector of trypanosomes (Hayes *et al*., 2014; Paparini *et al*., 2014; Fermino *et al*., 2015).

In conclusion, the terrestrial leech *C. bilineata* contains five lineages of trypanosomes that are genetically and morphologically very closely related to *T. cyclops*. We propose *T. cyclops australiensis* as a subspecies of *T. cyclops cyclops* based on morphology, phylogenetic and biogeography considerations.

## Sequence data availability

Sequences of the 18S rDNA, GAPDH and ND5 loci derived from the trypanosomes described in this study have been submitted to GenBank and can be found under accession numbers MW872344 to MW872359 and MW874225 to MW874260.

## Taxonomic summary

*New subspecies description*: Phylum Euglenozoa, Cavalier-Smith, 1981; class Kinetoplastea, Honigberg, 1963; order Trypanosomatida (Kent, 1880) Hollande, 1952; family Trypanosomatidae, Doflein, 1951; *Trypanosoma cyclops cyclops* (Weinman, 1972).

*Hapantotype*: cultures from Macaques, *Macaca nemestrina*, West Malaysia, 1969 (ATCC 30282 and 30283) (Weinman, 1972).

*Paratypes*: cultures reported by Weinman from *Macaca ira* (now called *Macaca fascicularis*), Malaysia.

*Type host*: *Macaca nemestrina* and *Macaca ira*.

*Etymology*: *Trypanosoma cyclops* is assigned as the nominotypical subspecies *Trypanosoma cyclops cyclops* so that *Trypanosoma cyclops australiensis* can be adequately distinguished from it as a subspecies.

*Diagnosis*: DNA sequences unique to *T. cyclops cyclops* are deposited in the GenBank with accession numbers AJ131958.1, MW87424 and MW874260 for the 18S rDNA, GAPDH and ND5 loci.

*New subspecies description*: Phylum Euglenozoa, Cavalier-Smith, 1981; class Kinetoplastea, Honigberg, 1963; order Trypanosomatida (Kent, 1880) Hollande, 1952; family Trypanosomatidae, Doflein, 1951; *Trypanosoma cyclops australiensis* (Ellis, Barratt, Lee and Stark n. sp., 2021).

*Hapantotype*: culture *Trypanosoma* sp. *ABF* (Hamilton *et al*., 2005).

*Paratypes*: cultures reported in this study from *Chtonobdella bilineata* and locality of leech collection is from Sydney, NSW, Australia.

*Type host*: Macropodidae, *Wallabia*, *Wallabia bicolour*.

*Additional host*: Domanibdellidae, *Chtonobdella, Chtonobdella bilineata*.

*Locality*: Rosemead Rd, Hornsby, NSW, Australia (−33.700469, 151.085419).

*Morphology*: Trypanosomes grown in culture primarily as epimastigotes with a body length (not including the flagellum) typically 6.5–27 *μ*m long and 1.1–5.7 *μ*m at its widest point. The nucleus is usually centrally located in the body, and the kinetoplast is close by.

*Etymology*: the species is named following zoological nomenclature for a subspecies. The subspecies name was chosen based on the finding of the trypanosomes in an Australian leech species.

*Diagnosis*: DNA sequences unique to *T. cyclops australiensis* are deposited in the GenBank with accession numbers MW872344–MW872359, MW874225–MW874240, MW874243–MW874258 for the 18S rDNA, GAPDH and ND5 loci.

## Data Availability

DNA sequence data from this study are deposited in the GenBank with accession numbers MW872344–MW872359 and MW874225–MW874260.

## Supplementary material

For supplementary material accompanying this paper visit https://doi.org/10.1017/S0031182021000639. click here to view supplementary material
