# Supplementary material for: A new subspecies of Trypanosoma cyclops found in the Australian terrestrial leech Chtonobdella bilineata
Source: Parasitology. 2021 Apr 12;148(10):1125–36. doi: 10.1017/S0031182021000639 (PMC8311967; doi:10.1017/S0031182021000639)
Supplement: Supplementary file 1 [file S0031182021000639sup001.docx]

**Supplementary File 1**

This document summarises changes to the bioinformatics methods used on MiSeq data for leech analyses.

**Leech Blood Meal Analyses**

During the read mapping phase, reads were mapped to the human 18S rDNA (NR_146146.1) sequence. To capture sequences from vertebrates, the following parameters were selected during this mapping stage; 5% gaps, maximum gap size of 10, minimum overlap of 100 bases, similarity of 90%., maximum mismatches per read = 5%, maximum ambiguous bases = 1. All other parameters were identical to the trypanosome identity workflow. Also, the following parameters were changed during the de novo assembly phase: contigs only required coverage of 10 reads, because the contents of the blood meal represented the lowest proportion of reads (i.e. this part of the sample was poorly represented).

**Leech Identity**

The following parameters were modified. During the read mapping phase, reads were mapped to the following reference sequence from a leech 18S rDNA sequence: KT592372.1 from *C. bilineata* isolate AU72. This leech was selected based on preliminary analyses of the genus *Chtonobdella*. To restrict mapping to sequences from leeches, the following parameters were selected during this mapping stage; 5% gaps, maximum gap size of 5, minimum overlap of 100 bases, similarity of 95%., maximum mismatches per read = 5%, maximum ambiguous bases = 1. All other parameters were identical to the blood meal analysis. Also, the following parameters were changed during the *de novo* assembly phase; contigs required coverage of 500 reads, because of the substantial amount of coverage obtained for the host leech. This value was set high to ensure that sequencing errors were not assembled as a variant contig.
